# Upadacitinib-induced paradoxical face and scalp dermatitis: A case report of a novel sequela

**DOI:** 10.1177/2050313X231164271

**Published:** 2023-04-14

**Authors:** Elena Pastukhova, Alison Spurr, Quentin Nakonechny, Jennifer Lipson

**Affiliations:** 1 Faculty of Medicine, University of Ottawa, Ottawa, ON, Canada; 2 Division of Dermatology, The Ottawa Hospital, University of Ottawa, Ottawa, ON, Canada; 3 Dynacare Laboratories, Ottawa, ON, Canada

**Keywords:** Upadacitinib, Janus kinase inhibitor, dermatitis, adverse events

## Abstract

Atopic dermatitis is a chronic, pruritic inflammatory cutaneous condition that can carry significant morbidity. Severe or recalcitrant atopic dermatitis is often treated with immunosuppressants, biologics, or immune-modulating small molecule therapies. The Janus kinase–signal transducer and activator of transcription pathway is highly implicated in atopic dermatitis pathogenesis, and agents that inhibit Janus kinase signalling are new to the atopic dermatitis landscape. Upadacitinib is a JAK1 inhibitor that has a good safety and efficacy profile and is increasingly being prescribed for atopic dermatitis. We report a case of a 35-year-old male with extensive atopic dermatitis that initially improved significantly on upadacitinib, then after 6 months developed a severe crusted dermatitic eruption on the head favouring a seborrheic distribution. While the pathogenesis of this paradoxical reaction is unclear, this phenomenon may involve a shift to a more Th1/Th17-mediated immune response.

## Introduction

Atopic dermatitis (AD) is a common inflammatory dermatosis characterized by chronic pruritus and eczematous eruption. AD affects approximately 10% of adults worldwide.^1^ Severe cases of AD can have negative impacts on quality of life, psychological wellness, and social functioning.^2^ AD can further be complicated by superinfections, which can lead to significant morbidity.^3^ The mainstay of management for mild-to-moderate AD involves topical moisturizers, corticosteroids, and calcineurin inhibitors, whereas more severe cases may require phototherapy and systemic treatment, such as with immunosuppressants, biologics, or immune-modulating small molecule therapies, such as Janus kinase (JAK) inhibitors.^4^ Herein, we report a case of paradoxical face and scalp dermatitis secondary to upadacitinib for the treatment of extensive AD.

## Case report

**Figure 1.**
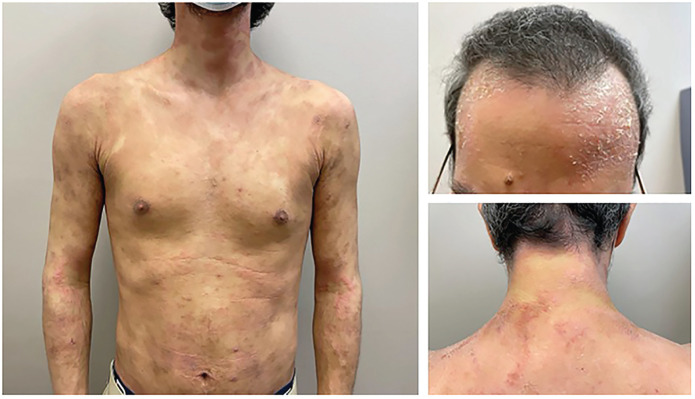
On initial consultation, there is significant erythema, scaling, and lichenification involving 70% body surface area (BSA), including the face, trunk, and extremities.

An otherwise healthy 35-year-old male was referred to dermatology with an 11-year history of poorly controlled AD. He described multiple episodes of major flares over his face, trunk, and extremities in the preceding decade requiring tapering courses of systemic corticosteroids as rescue therapy. Prior treatments included topical steroids, topical calcineurin inhibitors, and phototherapy, all with minimal improvement. On physical examination, there was significant erythema, scaling, and lichenification involving 70% body surface area (BSA), including the face, trunk, and extremities (Figure 1). At that time, his Eczema Area and Severity Index (EASI) score was 55 and Dermatology Life Quality Index (DLQI) was 26. He was started on methotrexate 15 mg po weekly with minimal improvement and side effects of malaise. This was subsequently discontinued due to lack of efficacy.

**Figure 2.**
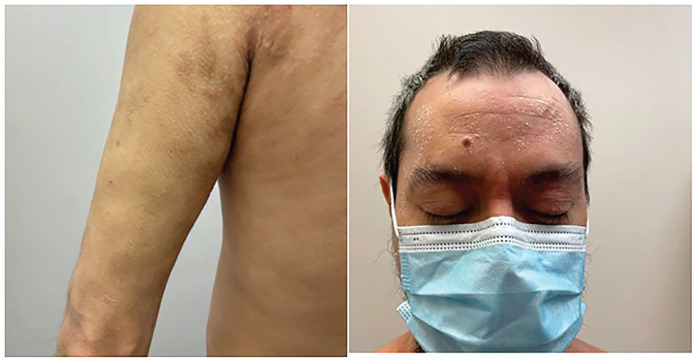
After 3 months of therapy, he had dramatic improvement of his pruritus and approximately 75% improvement in his skin with few residual eczematous patches and plaques on the face, neck, and arms.

In December 2021, the patient was started on upadacitinib 15 mg po daily for 1 month which was then increased to 30 mg po qd. After 3 months of therapy, he had dramatic improvement of his pruritus and approximately 75% improvement in his skin with few residual eczematous patches and plaques on the face, neck, and arms (Figure 2). He experienced no side effects, and his laboratory monitoring parameters were unremarkable. He was thrilled with the results.

**Figure 3.**
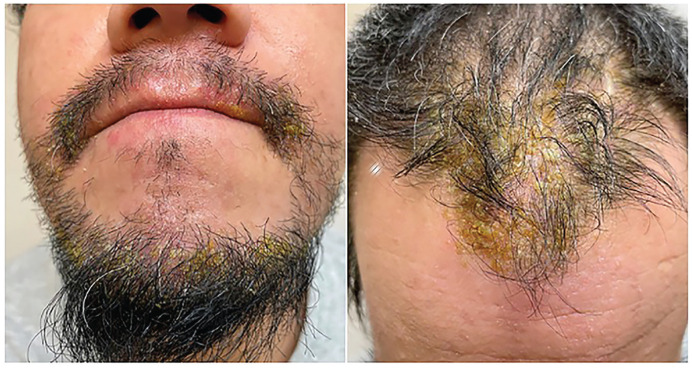
There are extensive erythematous patches and plaques with yellow exudative crusting involving the scalp, beard, and face.

**Figure 4.**
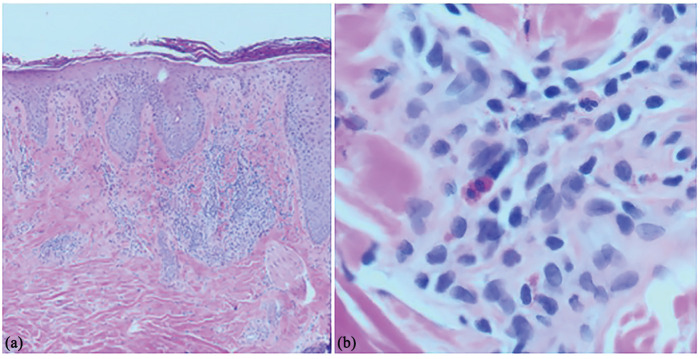
The punch biopsy of skin of the scalp shows irregular acanthosis with underlying papillary dermal fibrosis. There is a mixed inflammatory infiltrate within the papillary dermis with lymphocytes and eosinophils. The overlying stratum corneum shows orthokeratosis admixed with areas showing prominent neutrophilic involvement with a few neutrophils. Rare eosinophils are seen within the epidermis. (a) Punch biopsy of the scalp, hematoxylin and eosin (H&E) staining, magnification 40× and (b) punch biopsy of the scalp, H&E staining, magnification 400×.

In June 2022, the patient presented with a severe flare on the trunk and extremities, and a crusting exudative eruption on the scalp and face. On examination, there were extensive erythematous patches and plaques with yellow exudative crusting involving the scalp, beard, and face (Figure 3). He had tender left cervical lymphadenopathy. At that time, the differential included a flare of his AD with secondary infection or allergic contact dermatitis. He was started on amoxicillin-clavulanic acid and valacyclovir referred for patch testing, and a biopsy and cultures were performed. He continued to worsen despite antibiotics, antiviral, topical steroids, and upadacitinib. Viral and bacterial swabs were negative. The biopsy demonstrated spongiosis with eosinophilia, consistent with AD or possibly allergic contact dermatitis (Figure 4). Upadacitinib was held, and he was given a course of prednisone with good effect. In mid-July, following the resolution of this flare, he restarted upadacitinib 15 mg, and in early August, he developed a similar severe flare, this time, more limited to the face and scalp with oozing crusted plaques lasting until early September. Bacterial culture, viral culture, and polymerase chain reaction (PCR) were once again negative. His upadacitinib was held again, and he cleared with prednisone. The patient was optimistic that these severe flares were unrelated to upadacitinib, and thus once his inflammation subsided, he restarted the medication only to flare again requiring a third course of prednisone in October. A punch biopsy from the scalp during the final flare demonstrated irregular acanthosis with underlying lymphoeosinophilic inflammatory infiltrate with prominent neutrophil crusting. Period-acid Schiff and Grocott methenamine–silver staining were negative. No intraepidermal vesicular dermatitis or bacteria were identified. At this point, it was decided to permanently discontinue upadacitinib and change to dupilumab. Patch testing is pending at the time of publication.

## Discussion

The etiopathogenesis of AD is multifactorial, involving a complex interplay of genetic, environmental, and immunologic factors. Studies have implicated mutations in the *FLG-OVOL1-Interleukin-13* genetic axis as predisposing catalysts to promoting T-cell infiltration of the skin, facilitating dysbiosis, and chronic inflammation.^5^ The main immunologic pathway implicated in AD is a preponderant type 2 helper T-cell (Th2) response.^5^ Cytokines that are upregulated include interleukin (IL)-4, interferon (IFN)-γ, IL-13, IL-22, IL-23, and IL-31, which subsequently interact with type I or II cytokine plasma membrane receptors. JAKs are a family of cytoplasmic signal transduction proteins that bind to intracellular domains of cytokine receptors and propagate signalling cascades. JAK signalling results in the activation and nuclear translocation of signal transducers and activators of transcription (STATs), leading to transcription of pro-inflammatory genes, B-lymphocyte maturation, and eosinophil activation. Multiple JAKs, such as JAK1, JAK2, JAK3, and TYK2, have been studied, allowing for their selective targeting. Upadacitinib is an oral JAK1 inhibitor that is commonly used in moderate-to-severe AD.

Paradoxical cutaneous adverse events have previously been described with immunotherapy agents.^6^ Our patient tolerated upadacitinib well for approximately 6 months prior to developing a paradoxical worsening of AD in a seborrheic distribution, with improvement upon discontinuing upadacitinib, and recurrence upon re-starting the medication. Utilizing the validated Naranjo Scale^7^ for assessing causality of drug adverse events, we assigned the score of 9, which corresponds to a definite adverse reaction to upadacitinib (Table 1).

**Table 1.** Evaluation of head and neck dermatitis as an adverse effect of upadacitinib using the Naranjo Scale.^7^

Questions	Yes	No	Do not know	Score
Are there previous conclusive reports on this reaction?	+1	0	0	1
Did the adverse event appear after the suspected drug was administered?	+2	–1	0	1
Did the adverse reaction improve when the drug was discontinued, or a specific antagonist was administered?	+1	0	0	1
Did the adverse reaction re-appear when the drug was re-administered?	+2	–1	0	2
Are there alternative causes (other than the drug) that could on their own have caused the reaction?	–1	+2	0	2
Did the reaction reappear when a placebo was given?	–1	+2	0	0
Was the drug detected in the blood (or other fluids) in concentrations known to?	+1	0	0	0
Was the reaction more severe when the dose was increased, or less severed when the dose was decreased?	+1	0	0	0
Did the patient have a similar reaction to the same or similar drugs in any previous exposure?	+1	0	0	1
Was the adverse event confirmed by any objective evidence?	+1	0	0	1
Total score:				9

Results: ⩾9: definite; 5–8: probable; 1–4: possible; ⩽0: unlikely.

In the present patient, the notable seborrheic distribution of worsened dermatitis suggests that the reaction was either severe seborrheic dermatitis or sebopsoriasis which was supported by histopathology. Interestingly, one study demonstrated new onset of significant seborrheic dermatitis and sebopsoriasis in two patients on dupilumab, an IL-4/13 cytokine receptor inhibitor.^8^ IL-4 has a key role in inducing Th2 differentiation and JAK-STAT signalling cascade. A recent systematic review concluded that the worsening of head and neck dermatitis on dupilumab is secondary to Th2 inhibition that results in hyperproliferation of *Malassezia furfur* and increased Th1/Th17 signalling.^9^ We postulate a similar mechanism is involved in our patient, whereby JAK1 inhibition downregulates the Th2 axis and disrupts the homeostasis in T-cell differentiation pathways towards increased Th1/Th17 signalling and inflammation. In addition, upadacitinib may augment the skin microbiota, leading to *Malassezia* overgrowth and subsequent seborrhea. To the best of our knowledge, we present the first reported case of upadacitinib-induced head and neck dermatitis. Clinicians may consider this adverse event in patients on upadacitinib with recalcitrant eczematous dermatosis.
